# Supplementary material for: A Snapshot of European Children’s Eating Habits: Results from the Fourth Round of the WHO European Childhood Obesity Surveillance Initiative (COSI)
Source: Nutrients. 2020 Aug 17;12(8):2481. doi: 10.3390/nu12082481 (PMC7468747; doi:10.3390/nu12082481)
Supplement: Supplementary file 1 [file nutrients-12-02481-s001.pdf]

Supplementary Box S1. Description of national ethics approval for countries featured in this paper who were participating in the WHO European Childhood Obesity Surveillance Initiative (COSI) Round 4 (2015-2017)

Ethics approval for this study was provided by the following institutions: Albania (Scientific Committee of Institute of Public Health, Decision number: 953, 13 July 2015), Bulgaria (Commission of Medical Ethics at the National Center of Public Health and Analyses, Sofia, Bulgaria, Project identification code - 060 - МП 325 -68 COSI, 25 February 2016), Croatia (Ethical Committee of the Croatian Institute for Public Health, Registry number: 80-2660/1-15, 25 September 2015), Czechia (Ethical Committee of the Institute of Endocrinology, Prague, Czech Republic, AZV MZČR 17-31670 A, 20 June 2016), Denmark (Research and Innovation Organization, SDU, 10.829, 27/06/2016), Estonia (Tallinn Medical Research Ethics Committee, TMEK decision No 1376, 28 March 2016), Georgia (Bioethics Council at National Center for Disease Control and Public Health of Georgia, Project identification code: 2019-52, 4 November, 2019), Ireland (University College Dublin Human Research Ethics Committee – Sciences, Project identification code: LS-15-43-Heinen-Kelleher, Date of approval: 26th August 2015), Italy (National Institute of Health, Prot. PRE - 739/15, 10 November 2015), Kazakhstan (Local Ethics Committee, approval for Scientific and technical program "Development and implementation of modern technologies for healthy lifestyle promotion and prevention of diseases based on the study of non-medical determinants of health among children", 2015), Kyrgyzstan (The Ethics Committee on compliance of research to ethical norms for medical research, Project identification code: No.1/1, Date of approval: 22 February 2018), Latvia (Central Medical Ethics Committee, Project identification code: 01-29.1/6, Date of approval: 25 September 2015), Lithuania (Name of the ethics committee: Lithuanian Bioethics Committee (Lietuvos bioetikos komitetas); Project identification code: 08-02-19; 19 February 2008. After the approval we received renewal of a bioethics authorization in 2010 (on 2010-01-04), 2013 (on 2013 -01-09) and 2019 (on 2019-03-12), Montenegro (Ethical Committee of the Institute of Public Health of Montenegro Project identification code: WHO 2016/627456-0, Date of approval: 28th April 2020), Poland (Bioethics Committee of the Institute of Mother and Child, Warsaw, Poland, Project identification number: 22/2015, 26 November 2015), Portugal (National Commission of Data Protection; Aut nº5418/2016 for all rounds of COSI Portugal, 7 June 2016), Romania (Intern Ethical Committee of the National Institute of Public Health, Romania , Project identification code: WHO 2016/650301-0, Date of approval: 6 April 2016), Russian Federation (National Institute of Health., Prot. PRE - 739/15, November 2015 National Institute of Health, Prot. PRE - 739/15, November 2015), Tajikistan (Ministry of Health and Social Protection of Tajikistan, Project identification code: #858, Date of approval: 18 November 2016), Turkey (Kecioren Training and Research Hospital, Clinical Researches Ethics Committee; Approval Date: 26.10.2016, Project identification code: Health System Strengthening and Support Project, LN: 8531-TR, Date of Approval 2015: L.2.12. Obesity Fighting Project , Sub Component 1.1, L.2.11). In Turkmenistan, ethics approval was granted from the Ministry of Healthcare and Medical Industry (MOHMI). Malta did not go through Ethics Committee as all the work involved in COSI data collection and analysis is part and parcel of the existing School Health Service, which is an ongoing process. Data for COSI in Spain was collected as part of the ALADINO Spanish study, which didn't ask for an ethics committee approval, since it is not mandatory in Spain. However, the Principal Investigators confirm that the study was conducted in accordance with the Declaration of Helsinki and all parents/guardians of subjects participating gave their informed consent for inclusion before they participated in the study.

Supplementary Table S1. Frequency of breakfast consumption among boys and girls, by country. COSI/WHO Europe Round 4 (2015-17)

Country	*Boys (%)*				*Girls (%)*				*Total (%)*			
	Never	*Some days (1-3days)*	*Most days (4-6 days)*	*Every day*	*Never*	*Some days (1-3 days)*	*Most days (4-6 days)*	*Every day*	*Never*	*Some days (1-3 days)*	*Most days (4-6 days)*	*Every day*
Eastern Europe												
BULGARIA	*1.5*	*11.4*	*10.0*	*77.1*	*1.8*	*9.9*	*9.7*	*78.7*	*1.7*	*10.7*	*9.8*	*77.8*
CZECHIA	*1.2*	*8.7*	*12.5*	*77.7*	*1.3*	*10.5*	*10.0*	*78.2*	*1.2*	*9.5*	*11.3*	*77.9*
POLAND	*1.2*	*6.5*	*7.0*	*85.3*	*0.8*	*6.8*	*8.7*	*83.7*	*1.0*	*6.6*	*7.8*	*84.5*
ROMANIA	*7.1*	*19.1*	*9.4*	*64.4*	*6.5*	*20.6*	*9.0*	*63.8*	*6.8*	*19.8*	*9.2*	*64.1*
RUSSIAN FEDERATION	*0.3*	*2.8*	*3.9*	*92.9*	*0.5*	*2.2*	*4.7*	*92.6*	*0.4*	*2.5*	*4.3*	*92.8*
Northern Europe												
DENMARK	*0*	*0.9*	*3.0*	*96.0*	*0.2*	*2.2*	*3.0*	*94.7*	*0.1*	*1.5*	*3.0*	*95.4*
IRELAND	*0.6*	*2.6*	*4.1*	*92.8*	*0.3*	*1.9*	*5.2*	*92.7*	*0.4*	*2.2*	*4.6*	*92.7*
LITHUANIA	*5.1*	*28.3*	*10.8*	*55.8*	*5.2*	*28.3*	*11.5*	*55.0*	*5.1*	*28.3*	*11.2*	*55.4*
LATVIA[a]	*1.9*	*11.1*	*11.2*	*75.8*	*2.7*	*13.3*	*12.2*	*71.8*	*2.3*	*12.2*	*11.7*	*73.7*
Southern Europe												
ALBANIA	*3.7*	*11.4*	*8.5*	*76.4*	*3.9*	*12.5*	*10.3*	*73.2*	*3.8*	*11.9*	*9.4*	*74.9*
CROATIA	*1.1*	*9.7*	*11.4*	*77.9*	*1.6*	*8.9*	*13.6*	*75.8*	*1.4*	*9.3*	*12.5*	*76.9*
ITALY	*n.a.*	*n.a.*	*n.a.*	*n.a.*	*n.a.*	*n.a.*	*n.a.*	*n.a.*	*n.a.*	*n.a.*	*n.a.*	*n.a.*
MALTA [b]	*7.1*	*20.4*	*14.3*	*58.2*	*7.7*	*21.7*	*14.4*	*56.2*	*7.4*	*21.0*	*14.3*	*57.2*
MONTENEGRO [a]	*0.4*	*2.0*	*4.9*	*92.8*	*0.6*	*3.6*	*4.8*	*91.0*	*0.5*	*2.7*	*4.8*	*92.0*
PORTUGAL	*0.4*	*1.4*	*1.4*	*96.9*	*0.6*	*1.5*	*2.0*	*96.0*	*0.5*	*1.4*	*1.7*	*96.4*
SAN MARINO	*n.a.*	*n.a.*	*n.a.*	*n.a.*	*n.a.*	*n.a.*	*n.a.*	*n.a.*	*n.a.*	*n.a.*	*n.a.*	*n.a.*
SPAIN	*0.7*	*3.1*	*3.1*	*93.0*	*0.4*	*2.7*	*4.2*	*92.7*	*0.6*	*2.9*	*3.7*	*92.8*
Central Asia												
KAZAKHSTAN	*2.0*	*12.9*	*34.8*	*50.4*	*1.8*	*15.0*	*35.9*	*47.3*	*1.9*	*14.0*	*35.3*	*48.9*
KYRGYZSTAN [a]	*5.3*	*14.3*	*9.2*	*71.2*	*4.7*	*15.4*	*11.4*	*68.5*	*5.0*	*14.8*	*10.3*	*69.9*

TAJIKISTAN	*1.4*	*14.5*	*9.1*	*75.0*	*1.1*	*13.0*	*9.7*	*76.1*	*1.3*	*13.8*	*9.4*	*75.5*
TURKMENISTAN	*0*	*3.7*	*7.0*	*89.4*	*0*	*3.0*	*7.2*	*89.8*	*0*	*3.3*	*7.1*	*89.6*
Western Asia												
GEORGIA	*1.7*	*19.6*	*13.5*	*65.2*	*2.3*	*22.4*	*13.4*	*61.9*	*2.0*	*21.0*	*13.4*	*63.6*
TURKEY	*3.0*	*6.7*	*11.5*	*78.8*	*2.8*	*7.5*	*11.4*	*78.3*	*2.9*	*7.1*	*11.5*	*78.5*
Pooled estimates c	**2.4**	**8.4**	**10.2**	**79.0**	**2.1**	**8.7**	**10.7**	**78.5**	**2.3**	**8.6**	**10.4**	**78.8**

Data on frequency consumption of breakfast was not collected in Italy and San Marino.

a Statistically significant difference of proportions between boys and girls - Pearson's chi-squared corrected using Rao-Scott method, $p < 0.05$.

b Statistically significant difference of proportions between boys and girls - Pearson's chi-squared corrected using Rao-Scott method, $p < 0.005$.

c Pooled values were estimated including the following age groups/countries: 7-year-olds from Bulgaria, Czechia, Denmark, Kyrgyzstan, Georgia, Ireland, Latvia, Lithuania, Malta, Montenegro, Portugal, Spain, Tajikistan, Turkey and Turkmenistan; 8-year-olds from Albania, Croatia, Poland and Romania; and 9-year-olds from Kazakhstan.

Supplementary Table S2 – Frequency of fresh fruit consumption among boys and girls, by country. COSI/WHO Europe Round 4 (2015-17)

Country	*Boys (%)*				*Girls (%)*				*Total (%)*			
	Never or less than once a week	*Some days (1-3 days)*	*Most days (4-6 days)*	*Every day*	*Never or less than once a week*	*Some days (1-3 days)*	*Most days (4-6 days)*	*Every day*	*Never or less than once a week*	*Some days (1-3 days)*	*Most days (4-6 days)*	*Every day*
Eastern Europe												
BULGARIA	*5.2*	*31.2*	*28.8*	*34.8*	*5.5*	*28.1*	*30.3*	*36.1*	*5.4*	*29.7*	*29.5*	*35.4*
CZECHIA	*0.9*	*19.9*	*37.9*	*41.4*	*0.6*	*16.8*	*35.8*	*46.7*	*0.7*	*18.4*	*36.9*	*44.0*
POLAND	*6.2*	*26.9*	*31.4*	*35.5*	*3.8*	*27.2*	*31.5*	*37.6*	*5.0*	*27.0*	*31.4*	*36.6*
ROMANIA [a]	*7.5*	*27.7*	*23.4*	*41.4*	*5.0*	*26.8*	*25.5*	*42.7*	*6.2*	*27.3*	*24.5*	*42.1*
RUSSIAN FEDERATION	*2.9*	*15.7*	*32.2*	*49.2*	*2.0*	*14.5*	*31.2*	*52.3*	*2.4*	*15.1*	*31.7*	*50.7*
Northern Europe												
DENMARK	*2.7*	*12.3*	*27.7*	*57.3*	*2.8*	*8.2*	*26.3*	*62.7*	*2.7*	*10.4*	*27.1*	*59.8*
IRELAND	*6.7*	*13.7*	*18.9*	*60.7*	*4.5*	*11.4*	*22.5*	*61.6*	*5.7*	*12.6*	*20.6*	*61.1*
LITHUANIA [b]	*10.1*	*37.3*	*34.7*	*18.0*	*7.4*	*33.2*	*39.0*	*20.4*	*8.8*	*35.3*	*36.8*	*19.2*
LATVIA [b]	*7.2*	*36.4*	*33.6*	*22.8*	*5.1*	*35.0*	*36.5*	*23.5*	*6.1*	*35.7*	*35.1*	*23.2*
Southern Europe												
ALBANIA	*4.7*	*17.1*	*21.0*	*57.2*	*3.5*	*17.2*	*19.6*	*59.6*	*4.1*	*17.2*	*20.4*	*58.4*
CROATIA [b]	*7.2*	*30.0*	*29.1*	*33.8*	*3.6*	*28.6*	*34.0*	*33.7*	*5.4*	*29.3*	*31.5*	*33.8*
ITALY [c]	*7.1*	*n.a.*	*n.a.*	*71.5*	*5.6*	*n.a.*	*n.a.*	*73.7*	*6.4*	*n.a.*	*n.a.*	*72.6*
MALTA [c]	*8.7*	*26.6*	*20.1*	*44.5*	*5.1*	*28.1*	*23.0*	*43.9*	*6.9*	*27.4*	*21.5*	*44.2*
MONTENEGRO [b]	*3.0*	*17.9*	*32.5*	*46.6*	*1.3*	*16.4*	*37.5*	*44.7*	*2.2*	*17.2*	*34.9*	*45.7*
PORTUGAL	*3.8*	*14.7*	*19.3*	*62.2*	*2.8*	*14.0*	*19.3*	*63.9*	*3.3*	*14.3*	*19.3*	*63.1*
SAN MARINO [b]	*2.2*	*n.a.*	*n.a.*	*83.2*	*3.6*	*n.a.*	*n.a.*	*78.8*	*3.0*	*n.a.*	*n.a.*	*80.8*
SPAIN	*8.6*	*30.0*	*32.9*	*28.6*	*7.4*	*29.4*	*33.9*	*29.3*	*8.0*	*29.7*	*33.4*	*28.9*
Central Asia												

KAZAKHSTAN	*9.5*	*35.0*	*21.9*	*33.5*	*8.4*	*30.8*	*27.8*	*33.0*	*9.0*	*32.9*	*24.8*	*33.3*
KYRGYZSTAN	*22.9*	*42.0*	*17.2*	*18.0*	*22.4*	*41.3*	*18.0*	*18.3*	*22.6*	*41.7*	*17.6*	*18.1*
TAJIKISTAN	*20.9*	*30.5*	*13.8*	*34.8*	*21.9*	*30.9*	*15.1*	*32.2*	*21.4*	*30.7*	*14.4*	*33.5*
TURKMENISTAN	*2.7*	*5.1*	*22.5*	*69.7*	*2.4*	*6.0*	*21.2*	*70.4*	*2.5*	*5.6*	*21.9*	*70.1*
Western Asia												
GEORGIA	*9.9*	*28.1*	*39.0*	*22.9*	*10.1*	*27.4*	*38.0*	*24.5*	*10.0*	*27.8*	*38.5*	*23.7*
TURKEY [a]	*6.8*	*19.4*	*24.4*	*49.4*	*5.5*	*17.8*	*24.4*	*52.3*	*6.1*	*18.6*	*24.4*	*50.8*
Pooled estimates [b,d]	**8.2**	**24.4**	**25.7**	**41.7**	**6.7**	**22.9**	**27.1**	**43.3**	**7.5**	**23.7**	**26.4**	**42.5**

For vegetable consumption in Italy and San Marino, the only data variables available were “every day” or “never or less than once a week.”

a Statistically significant difference of proportions between boys and girls - Pearson's chi-squared corrected using Rao-Scott method, $p < 0.05$.

b Statistically significant difference of proportions between boys and girls - Pearson's chi-squared corrected using Rao-Scott method, $p < 0.005$.

c Statistically significant difference of proportions between boys and girls - Pearson's chi-squared corrected using Rao-Scott method, $p < 0.0001$.

d Pooled values were estimated including the following age groups/countries: 7-year-olds from Bulgaria, Czechia, Denmark, Kyrgyzstan, Georgia, Ireland, Latvia, Lithuania, Malta, Montenegro, Portugal, Spain, Tajikistan, Turkey and Turkmenistan; 8-year-olds from Albania, Croatia, Poland and Romania; and 9-year-olds from Kazakhstan.

Supplementary Table S3 – Frequency of vegetable consumption among boys and girls, by country. COSI/WHO Europe Round 4 (2015-17)

Country	*Boys (%)*				*Girls (%)*				*Total (%)*			
	Never or less than once a week	*Some days (1-3 days)*	*Most days (4-6 days)*	*Every day*	*Never or less than once a week*	*Some days (1-3 days)*	*Most days (4-6 days)*	*Every day*	*Never or less than once a week*	*Some days (1-3 days)*	*Most days (4-6 days)*	*Every day*
Eastern Europe												
BULGARIA	*8.7*	*32.3*	*33.4*	*25.5*	*7.5*	*30.2*	*33.3*	*29.0*	*8.2*	*31.3*	*33.4*	*27.2*
CZECHIA	*1.3*	*32.3*	*36.2*	*30.2*	*1.4*	*24.6*	*38.1*	*35.8*	*1.3*	*28.5*	*37.2*	*33.0*
POLAND	*11.5*	*36.6*	*30.2*	*21.7*	*10.3*	*33.8*	*31.3*	*24.6*	*10.9*	*35.2*	*30.7*	*23.1*
ROMANIA	*14.1*	*36.0*	*22.8*	*27.2*	*11.6*	*35.0*	*26.7*	*26.7*	*12.8*	*35.5*	*24.7*	*26.9*
RUSSIAN FEDERATION	*5.0*	*22.5*	*36.5*	*35.9*	*4.1*	*22.2*	*38.8*	*34.9*	*4.6*	*22.4*	*37.7*	*35.4*
Northern Europe												
DENMARK	*4.0*	*16.0*	*27.1*	*52.9*	*5.1*	*15.8*	*27.7*	*51.5*	*4.5*	*15.9*	*27.4*	*52.2*
IRELAND	*9.3*	*14.5*	*30.8*	*45.5*	*6.5*	*19.0*	*27.7*	*46.8*	*8.0*	*16.6*	*29.3*	*46.1*
LITHUANIA	*18.6*	*37.3*	*30.4*	*13.7*	*15.9*	*36.9*	*32.6*	*14.6*	*17.3*	*37.1*	*31.5*	*14.1*
LATVIA	*9.6*	*39.5*	*33.8*	*17.1*	*9.2*	*38.5*	*33.7*	*18.5*	*9.4*	*39.0*	*33.7*	*17.8*
Southern Europe												
ALBANIA	*10.9*	*33.7*	*28.5*	*26.9*	*8.7*	*31.9*	*32.6*	*26.8*	*9.8*	*32.9*	*30.5*	*26.8*
CROATIA	*9.1*	*41.3*	*33.5*	*16.1*	*6.9*	*42.1*	*33.6*	*17.4*	*8.0*	*41.7*	*33.6*	*16.7*
ITALY [c]	*13.3*	*n.a.*	*n.a.*	*51.1*	*10.0*	*n.a.*	*n.a.*	*56.8*	*11.7*	*n.a.*	*n.a.*	*53.9*
MALTA [c]	*16.3*	*48.0*	*19.8*	*16.0*	*14.0*	*48.1*	*22.6*	*15.3*	*15.1*	*48.0*	*21.2*	*15.6*
MONTENEGRO [a]	*6.0*	*26.6*	*37.9*	*29.6*	*3.3*	*28.0*	*39.0*	*29.7*	*4.7*	*27.3*	*38.4*	*29.7*
PORTUGAL	*7.7*	*26.5*	*29.1*	*36.8*	*6.4*	*25.0*	*30.0*	*38.6*	*7.1*	*25.7*	*29.5*	*37.7*
SAN MARINO [c]	*8.0*	*n.a.*	*n.a.*	*70.3*	*4.2*	*n.a.*	*n.a.*	*77.7*	*5.9*	*n.a.*	*n.a.*	*74.3*
SPAIN	*14.8*	*49.3*	*27.5*	*8.4*	*15.1*	*50.0*	*25.1*	*9.8*	*15.0*	*49.6*	*26.3*	*9.1*
Central Asia												
KAZAKHSTAN	*8.9*	*26.8*	*35.5*	*28.7*	*9.0*	*24.2*	*35.1*	*31.7*	*8.9*	*25.5*	*35.3*	*30.2*
KYRGYZSTAN	*16.5*	*31.1*	*22.3*	*30.0*	*15.4*	*30.8*	*21.6*	*32.2*	*16.0*	*31.0*	*22.0*	*31.1*

TAJIKISTAN	*15.1*	*23.5*	*17.6*	*43.8*	*14.9*	*25.2*	*17.6*	*42.4*	*15.0*	*24.3*	*17.6*	*43.1*
TURKMENISTAN	*1.0*	*7.9*	*21.9*	*69.2*	*1.7*	*8.1*	*23.2*	*67.0*	*1.4*	*8.0*	*22.5*	*68.1*
Western Asia												
GEORGIA	*17.5*	*33.1*	*34.8*	*14.6*	*16.7*	*33.4*	*35.7*	*14.2*	*17.1*	*33.2*	*35.2*	*14.4*
TURKEY	*23.7*	*41.6*	*23.4*	*11.3*	*17.0*	*43.1*	*27.5*	*12.5*	*20.4*	*42.3*	*25.4*	*11.9*
Pooled estimates [b,d]	***15.3***	***36.7***	***26.4***	***21.6***	***12.7***	***36.2***	***27.6***	***23.5***	***14.0***	***36.4***	***27.0***	***22.6***

For vegetable consumption in Italy and San Marino, the only data variables available were “every day” or “never or less than once a week.”

a Statistically significant difference of proportions between boys and girls - Pearson's chi-squared corrected using Rao-Scott method, $p < 0.05$.

b Statistically significant difference of proportions between boys and girls - Pearson's chi-squared corrected using Rao-Scott method, $p < 0.005$.

c Statistically significant difference of proportions between boys and girls - Pearson's chi-squared corrected using Rao-Scott method, $p < 0.0001$.

d Pooled values were estimated including the following age groups/countries: 7-year-olds from Bulgaria, Czechia, Denmark, Kyrgyzstan, Georgia, Ireland, Latvia, Lithuania, Malta, Montenegro, Portugal, Spain, Tajikistan, Turkey and Turkmenistan; 8-year-olds from Albania, Croatia, Poland and Romania; and 9-year-olds from Kazakhstan.

Supplementary Table S4 – Frequency of savory snack consumption among boys and girls, by country. COSI/WHO Europe Round 4 (2015-17)

Country	*Boys (%)*				*Girls (%)*				*Total (%)*			
	Never or less than once a week	*Some days (1-3 days)*	*Most days (4-6 days)*	*Every day*	*Never or less than once a week*	*Some days (1-3 days)*	*Most days (4-6 days)*	*Every day*	*Never or less than once a week*	*Some days (1-3 days)*	*Most days (4-6 days)*	*Every day*
Eastern Europe												
BULGARIA	*44.2*	*29.4*	*12.5*	*13.8*	*46.0*	*29.2*	*12.9*	*11.9*	*45.1*	*29.3*	*12.7*	*12.9*
CZECHIA	*26.7*	*70.5*	*2.6*	*0.3*	*30.2*	*67.6*	*1.7*	*0.5*	*28.4*	*69.1*	*2.1*	*0.4*
POLAND	*76.4*	*19.1*	*3.0*	*1.5*	*78.5*	*18.7*	*2.1*	*0.7*	*77.5*	*18.9*	*2.5*	*1.1*
ROMANIA [a]	*47.1*	*30.5*	*10.9*	*11.5*	*51.7*	*27.5*	*11.0*	*9.8*	*49.4*	*29.0*	*11.0*	*10.6*
RUSSIAN FEDERATION	*90.0*	*8.3*	*1.4*	*0.3*	*91.7*	*7.4*	*0.9*	*0*	*90.8*	*7.9*	*1.2*	*0.1*
Northern Europe												
DENMARK	*73.5*	*25.9*	*0.6*	*0*	*75.8*	*23.8*	*0.4*	*0*	*74.6*	*24.9*	*0.5*	*0*
IRELAND [a]	*54.6*	*38.9*	*5.4*	*1.1*	*42.2*	*48.8*	*7.2*	*1.9*	*48.7*	*43.5*	*6.2*	*1.5*
LITHUANIA	*82.3*	*14.4*	*2.8*	*0.6*	*83.9*	*13.0*	*2.5*	*0.6*	*83.1*	*13.7*	*2.6*	*0.6*
LATVIA [a]	*78.2*	*18.4*	*2.7*	*0.7*	*81.9*	*15.3*	*2.3*	*0.5*	*80.2*	*16.8*	*2.5*	*0.6*
Southern Europe												
ALBANIA	*40.9*	*25.0*	*12.7*	*21.4*	*40.3*	*24.9*	*13.4*	*21.5*	*40.6*	*24.9*	*13.0*	*21.5*
CROATIA	*50.0*	*38.5*	*7.2*	*4.3*	*50.9*	*37.3*	*8.8*	*3.1*	*50.4*	*37.9*	*8.0*	*3.7*
ITALY	*n.a.*	*n.a.*	*n.a.*	*n.a.*	*n.a.*	*n.a.*	*n.a.*	*n.a.*	*n.a.*	*n.a.*	*n.a.*	*n.a.*
MALTA [b]	*8.3*	*76.4*	*10.7*	*4.5*	*7.1*	*78.8*	*9.9*	*4.3*	*7.7*	*77.6*	*10.3*	*4.4*
MONTENEGRO	*40.1*	*35.2*	*15.2*	*9.6*	*42.9*	*36.2*	*12.6*	*8.4*	*41.4*	*35.6*	*14.0*	*9.0*
PORTUGAL	*84.8*	*13.2*	*1.6*	*0.3*	*85.0*	*13.0*	*1.6*	*0.4*	*84.9*	*13.1*	*1.6*	*0.3*
SAN MARINO	*n.a.*	*n.a.*	*n.a.*	*n.a.*	*n.a.*	*n.a.*	*n.a.*	*n.a.*	*n.a.*	*n.a.*	*n.a.*	*n.a.*
SPAIN	*69.4*	*27.0*	*2.7*	*0.9*	*70.6*	*26.1*	*2.6*	*0.7*	*70.0*	*26.5*	*2.7*	*0.8*
Central Asia												
KAZAKHSTAN	*61.2*	*25.2*	*7.0*	*6.6*	*64.5*	*21.8*	*7.8*	*5.8*	*62.8*	*23.5*	*7.4*	*6.2*
KYRGYZSTAN	*47.5*	*32.4*	*11.7*	*8.3*	*47.6*	*32.7*	*11.8*	*7.9*	*47.6*	*32.5*	*11.8*	*8.1*

TAJIKISTAN	*46.0*	*30.2*	*12.4*	*11.4*	*45.1*	*30.4*	*13.2*	*11.2*	*45.6*	*30.3*	*12.8*	*11.3*
TURKMENISTAN	*54.9*	*25.8*	*11.1*	*8.1*	*53.1*	*25.3*	*11.7*	*9.8*	*54.0*	*25.6*	*11.4*	*9.0*
Western Asia												
GEORGIA [a]	*56.4*	*24.5*	*16.2*	*3.0*	*57.4*	*26.9*	*12.8*	*2.9*	*56.9*	*25.6*	*14.5*	*3.0*
TURKEY	*49.6*	*29.6*	*12.9*	*7.8*	*51.8*	*29.2*	*12.7*	*6.3*	*50.7*	*29.4*	*12.8*	*7.1*
Pooled estimates [c]	***57.5***	***28.6***	***8.4***	***5.5***	***58.3***	***28.4***	***8.5***	***4.8***	***57.9***	***28.5***	***8.4***	***5.2***

Data on frequency consumption of savoury snack was not collected in Italy and San Marino.

a Statistically significant difference of proportions between boys and girls - Pearson's chi-squared corrected using Rao-Scott method, $p < 0.05$.

b Statistically significant difference of proportions between boys and girls - Pearson's chi-squared corrected using Rao-Scott method, $p < 0.0001$.

c Pooled values were estimated including the following age groups/countries: 7-year-olds from Bulgaria, Czechia, Denmark, Kyrgyzstan, Georgia, Ireland, Latvia, Lithuania, Malta, Montenegro, Portugal, Spain, Tajikistan, Turkey and Turkmenistan; 8-year-olds from Albania, Croatia, Poland and Romania; and 9-year-olds from Kazakhstan.

Supplementary Table S5 – Frequency of sweet snack consumption among boys and girls, by country. COSI/WHO Europe Round 4 (2015-17)

Country	*Boys (%)*				*Girls (%)*				*Total (%)*			
	Never or less than once a week	*Some days (1-3 days)*	*Most days (4-6 days)*	*Every day*	*Never or less than once a week*	*Some days (1-3 days)*	*Most days (4-6 days)*	*Every day*	*Never or less than once a week*	*Some days (1-3 days)*	*Most days (4-6 days)*	*Every day*
Eastern Europe												
BULGARIA [a]	*11.7*	*39.0*	*25.3*	*24.0*	*14.5*	*37.3*	*26.6*	*21.6*	*13.1*	*38.2*	*25.9*	*22.8*
CZECHIA	*5.8*	*76.1*	*14.7*	*3.3*	*7.5*	*77.0*	*12.9*	*2.5*	*6.7*	*76.5*	*13.8*	*2.9*
POLAND	*24.5*	*46.4*	*19.9*	*9.2*	*27.5*	*44.5*	*19.0*	*9.0*	*26.0*	*45.5*	*19.5*	*9.1*
ROMANIA	*23.7*	*38.0*	*19.5*	*18.9*	*23.2*	*38.3*	*18.9*	*19.6*	*23.4*	*38.2*	*19.2*	*19.3*
RUSSIAN FEDERATION	*23.3*	*44.7*	*23.1*	*8.9*	*23.6*	*43.7*	*24.5*	*8.2*	*23.4*	*44.2*	*23.8*	*8.5*
Northern Europe												
DENMARK	*21.3*	*73.5*	*4.7*	*0.6*	*26.7*	*69.3*	*3.8*	*0.2*	*23.9*	*71.5*	*4.2*	*0.4*
IRELAND	*25.3*	*50.4*	*17.0*	*7.3*	*20.3*	*52.8*	*19.8*	*7.1*	*22.9*	*51.5*	*18.3*	*7.2*
LITHUANIA [b]	*31.6*	*46.0*	*16.8*	*5.7*	*28.8*	*43.6*	*20.3*	*7.2*	*30.2*	*44.8*	*18.5*	*6.4*
LATVIA	*21.4*	*47.0*	*20.8*	*10.8*	*19.8*	*48.5*	*20.6*	*11.1*	*20.5*	*47.8*	*20.7*	*11.0*
Southern Europe												
ALBANIA	*29.0*	*33.6*	*17.8*	*19.6*	*29.0*	*32.8*	*18.0*	*20.1*	*29.0*	*33.2*	*17.9*	*19.8*
CROATIA	*20.3*	*48.5*	*18.5*	*12.7*	*20.8*	*49.1*	*18.6*	*11.6*	*20.5*	*48.8*	*18.5*	*12.2*
ITALY	*n.a.*	*n.a.*	*n.a.*	*n.a.*	*n.a.*	*n.a.*	*n.a.*	*n.a.*	*n.a.*	*n.a.*	*n.a.*	*n.a.*
MALTA [c]	*4.6*	*59.8*	*20.4*	*15.2*	*3.3*	*62.0*	*19.9*	*14.8*	*3.9*	*60.9*	*20.1*	*15.0*
MONTENEGRO	*18.9*	*43.0*	*24.7*	*13.4*	*18.5*	*45.5*	*21.0*	*15.0*	*18.7*	*44.2*	*23.0*	*14.1*
PORTUGAL	*68.4*	*26.0*	*4.2*	*1.3*	*67.2*	*26.4*	*4.3*	*2.1*	*67.8*	*26.2*	*4.3*	*1.7*
SAN MARINO	*n.a.*	*n.a.*	*n.a.*	*n.a.*	*n.a.*	*n.a.*	*n.a.*	*n.a.*	*n.a.*	*n.a.*	*n.a.*	*n.a.*
SPAIN [a]	*58.1*	*33.7*	*6.1*	*2.0*	*55.2*	*36.9*	*6.4*	*1.4*	*56.7*	*35.3*	*6.3*	*1.7*
Central Asia												
KAZAKHSTAN	*41.7*	*36.4*	*14.1*	*7.8*	*38.3*	*39.7*	*14.7*	*7.2*	*40.0*	*38.0*	*14.4*	*7.5*
KYRGYZSTAN	*23.7*	*35.7*	*21.5*	*19.1*	*22.3*	*35.9*	*22.8*	*19.0*	*23.0*	*35.8*	*22.1*	*19.0*

TAJIKISTAN	*32.2*	*36.6*	*16.1*	*15.2*	*30.5*	*38.9*	*15.4*	*15.1*	*31.4*	*37.7*	*15.8*	*15.2*
TURKMENISTAN	*24.8*	*31.8*	*21.9*	*21.5*	*24.3*	*31.4*	*23.7*	*20.6*	*24.5*	*31.6*	*22.8*	*21.1*
Western Asia												
GEORGIA	*11.1*	*26.7*	*42.9*	*19.3*	*11.4*	*26.8*	*43.7*	*18.1*	*11.3*	*26.7*	*43.3*	*18.7*
TURKEY [a]	*31.2*	*35.3*	*20.5*	*13.0*	*30.7*	*38.4*	*19.7*	*11.2*	*30.9*	*36.8*	*20.1*	*12.1*
Pooled estimates[d]	***33.2***	***39.2***	***16.8***	***10.8***	***32.3***	***40.6***	***17.3***	***9.8***	***32.8***	***39.9***	***17.0***	***10.3***

Data on frequency consumption of savoury snack was not collected in Italy and San Marino.

a Statistically significant difference of proportions between boys and girls - Pearson's chi-squared corrected using Rao-Scott method, $p < 0.05$.

b Statistically significant difference of proportions between boys and girls - Pearson's chi-squared corrected using Rao-Scott method, $p < 0.005$.

c Statistically significant difference of proportions between boys and girls - Pearson's chi-squared corrected using Rao-Scott method, $p < 0.0001$.

d Pooled values were estimated including the following age groups/countries: 7-year-olds from Bulgaria, Czechia, Denmark, Kyrgyzstan, Georgia, Ireland, Latvia, Lithuania, Malta, Montenegro, Portugal, Spain, Tajikistan, Turkey and Turkmenistan; 8-year-olds from Albania, Croatia, Poland and Romania; and 9-year-olds from Kazakhstan.

Supplementary Table 6 – Frequency of soft drinks consumption among boys and girls, by country. COSI/WHO Europe Round 4 (2015-17)

Country	*Boys (%)*				*Girls (%)*				*Total (%)*			
	Never or less than once a week	*Some days (1-3 days)*	*Most days (4-6 days)*	*Every day*	*Never or less than once a week*	*Some days (1-3 days)*	*Most days (4-6 days)*	*Every day*	*Never or less than once a week*	*Some days (1-3 days)*	*Most days (4-6 days)*	*Every day*
Eastern Europe												
BULGARIA	*63.8*	*17.8*	*7.8*	*10.6*	*66.9*	*16.3*	*7.7*	*9.1*	*65.3*	*17.1*	*7.7*	*9.9*
CZECHIA	*17.9*	*48.9*	*19.0*	*14.3*	*18.6*	*53.6*	*15.8*	*12.0*	*18.2*	*51.2*	*17.4*	*13.2*
POLAND	*41.3*	*28.6*	*16.4*	*13.8*	*43.8*	*27.6*	*16.0*	*12.6*	*42.6*	*28.1*	*16.2*	*13.2*
ROMANIA [a]	*62.6*	*22.7*	*7.4*	*7.3*	*66.1*	*20.9*	*7.1*	*5.9*	*64.4*	*21.8*	*7.3*	*6.6*
RUSSIAN FEDERATION	*67.1*	*16.9*	*7.3*	*8.7*	*70.5*	*17.1*	*5.5*	*7.0*	*68.7*	*17.0*	*6.4*	*7.9*
Northern Europe												
DENMARK [a]	*50.9*	*38.8*	*7.1*	*3.1*	*57.1*	*37.6*	*4.3*	*1.1*	*53.8*	*38.3*	*5.8*	*2.1*
IRELAND	*88.0*	*10.8*	*0.5*	*0.6*	*88.0*	*10.3*	*1.5*	*0.2*	*88.0*	*10.6*	*1.0*	*0.4*
LITHUANIA [b]	*69.0*	*21.0*	*7.7*	*2.3*	*75.1*	*16.9*	*6.4*	*1.6*	*72.0*	*19.0*	*7.0*	*2.0*
LATVIA [a]	*60.0*	*27.4*	*8.5*	*4.2*	*63.9*	*24.6*	*7.4*	*4.1*	*62.0*	*26.0*	*7.9*	*4.1*
Southern Europe												
ALBANIA [a]	*58.1*	*26.0*	*7.6*	*8.3*	*63.8*	*23.3*	*7.4*	*5.5*	*60.8*	*24.7*	*7.5*	*6.9*
CROATIA	*37.6*	*31.1*	*15.0*	*16.3*	*40.0*	*32.6*	*14.5*	*12.8*	*38.8*	*31.9*	*14.8*	*14.6*
ITALY	*n.a.*	*n.a.*	*n.a.*	*n.a.*	*n.a.*	*n.a.*	*n.a.*	*n.a.*	*n.a.*	*n.a.*	*n.a.*	*n.a.*
MALTA [c]	*29.0*	*52.2*	*8.6*	*10.2*	*33.9*	*50.8*	*7.0*	*8.3*	*31.5*	*51.5*	*7.8*	*9.3*
MONTENEGRO [a]	*35.0*	*30.3*	*17.3*	*17.4*	*37.2*	*33.0*	*17.1*	*12.7*	*36.0*	*31.6*	*17.2*	*15.2*
PORTUGAL [a]	*58.5*	*25.5*	*8.0*	*8.0*	*61.4*	*25.2*	*7.7*	*5.8*	*59.9*	*25.4*	*7.8*	*6.9*
SAN MARINO	*n.a.*	*n.a.*	*n.a.*	*n.a.*	*n.a.*	*n.a.*	*n.a.*	*n.a.*	*n.a.*	*n.a.*	*n.a.*	*n.a.*
SPAIN	*83.1*	*13.3*	*2.5*	*1.1*	*84.9*	*11.4*	*2.7*	*1.1*	*84.0*	*12.4*	*2.6*	*1.1*
Central Asia												
KAZAKHSTAN	*49.8*	*26.9*	*12.7*	*10.6*	*50.8*	*27.4*	*10.5*	*11.3*	*50.3*	*27.2*	*11.6*	*10.9*
KYRGYZSTAN	*40.7*	*30.8*	*13.6*	*14.9*	*40.3*	*32.3*	*12.6*	*14.8*	*40.5*	*31.5*	*13.1*	*14.8*

TAJIKISTAN	*34.8*	*22.8*	*10.5*	*31.9*	*32.7*	*20.9*	*12.5*	*33.8*	*33.8*	*21.9*	*11.5*	*32.8*
TURKMENISTAN	*34.4*	*22.1*	*16.2*	*27.3*	*33.9*	*24.5*	*17.2*	*24.3*	*34.1*	*23.3*	*16.7*	*25.8*
Western Asia												
GEORGIA [b]	*42.9*	*29.4*	*21.0*	*6.7*	*49.1*	*28.9*	*16.4*	*5.6*	*46.0*	*29.2*	*18.7*	*6.1*
TURKEY [b]	*51.4*	*28.9*	*11.5*	*8.1*	*56.2*	*28.0*	*8.9*	*6.9*	*53.8*	*28.5*	*10.2*	*7.5*
Pooled estimates	***54.4***	***25.3***	***10.4***	***9.9***	***56.6***	***24.9***	***9.6***	***8.9***	***55.4***	***25.1***	***10.0***	***9.4***

Data on frequency consumption of savoury snack was not collected in Italy and San Marino.

a Statistically significant difference of proportions between boys and girls - Pearson's chi-squared corrected using Rao-Scott method, $p < 0.05$.

b Statistically significant difference of proportions between boys and girls - Pearson's chi-squared corrected using Rao-Scott method, $p < 0.005$.

c Statistically significant difference of proportions between boys and girls - Pearson's chi-squared corrected using Rao-Scott method, $p < 0.0001$.

d Pooled values were estimated including the following age groups/countries: 7-year-olds from Bulgaria, Czechia, Denmark, Kyrgyzstan, Georgia, Ireland, Latvia, Lithuania, Malta, Montenegro, Portugal, Spain, Tajikistan, Turkey and Turkmenistan; 8-year-olds from Albania, Croatia, Poland and Romania; and 9-year-olds from Kazakhstan.

Supplementary Figure S1. Description of the WHO European Childhood Obesity Surveillance Initiative family form question related to child dietary habits, COSI Round 4 (2015-17)

(20) ***Over a typical or usual week, how often does your child eat or drink the following kinds of foods or beverages? Please tick one box for each line.***

	Never	Less than once a week	Some days (1-3 days)	Most days (4-6 days)	Every day
Fresh fruit	☐	☐	☐	☐	☐
Vegetables (excluding potatoes)	☐	☐	☐	☐	☐
100% fruit juice	☐	☐	☐	☐	☐
Soft drinks containing sugar	☐	☐	☐	☐	☐
Flavoured milk	☐	☐	☐	☐	☐
Diet or “light” soft drinks	☐	☐	☐	☐	☐
Low fat/ semi-skimmed milk	☐	☐	☐	☐	☐
Whole fat milk	☐	☐	☐	☐	☐
Cheese	☐	☐	☐	☐	☐
Yoghurt, milk pudding, cream cheese/quark or other dairy products	☐	☐	☐	☐	☐
Meat	☐	☐	☐	☐	☐
Fish	☐	☐	☐	☐	☐
Savoury snacks like potato crisps, corn chips, popcorn or peanuts	☐	☐	☐	☐	☐
Sweet treats like candy bar or chocolate	☐	☐	☐	☐	☐
Foods like biscuits, cakes, doughnuts or pies	☐	☐	☐	☐	☐
Foods like pizza, French fries, fried potatoes, hamburgers, sausages or meat pies	☐	☐	☐	☐	☐
